# Absence of Nrf2 or Its Selective Overexpression in Neurons and Muscle Does Not Affect Survival in ALS-Linked Mutant hSOD1 Mouse Models

**DOI:** 10.1371/journal.pone.0056625

**Published:** 2013-02-13

**Authors:** Marcelo R. Vargas, Neal C. Burton, Li Gan, Delinda A. Johnson, Matthias Schäfer, Sabine Werner, Jeffrey A. Johnson

**Affiliations:** 1 Division of Pharmaceutical Sciences, University of Wisconsin, Madison, Wisconsin, United States of America; 2 Waisman Center, University of Wisconsin, Madison, Wisconsin, United States of America; 3 Molecular and Environmental Toxicology Center, University of Wisconsin, Madison, Wisconsin, United States of America; 4 Center for Neuroscience, University of Wisconsin, Madison, Wisconsin, United States of America; 5 Department of Biology, Institute of Molecular Health Sciences, ETH Zurich, Honggerberg, Switzerland; University of Florida, United States Of America

## Abstract

The nuclear factor erythroid 2-related factor 2 (Nrf2) governs the expression of antioxidant and phase II detoxifying enzymes. Nrf2 activation can prevent or reduce cellular damage associated with several types of injury in many different tissues and organs. Dominant mutations in Cu/Zn-superoxide dismutase (SOD1) cause familial forms of amyotrophic lateral sclerosis (ALS), a fatal disorder characterized by the progressive loss of motor neurons and subsequent muscular atrophy. We have previously shown that Nrf2 activation in astrocytes delays neurodegeneration in ALS mouse models. To further investigate the role of Nrf2 in ALS we determined the effect of absence of Nrf2 or its restricted overexpression in neurons or type II skeletal muscle fibers on symptoms onset and survival in mutant hSOD1 expressing mice. We did not observe any detrimental effect associated with the lack of Nrf2 in two different mutant hSOD1 animal models of ALS. However, restricted Nrf2 overexpression in neurons or type II skeletal muscle fibers delayed disease onset but failed to extend survival in hSOD1^G93A^ mice. These results highlight the concept that not only the pharmacological target but also the cell type targeted may be relevant when considering a Nrf2-mediated therapeutic approach for ALS.

## Introduction

The nuclear factor erythroid 2-related factor 2 (Nrf2) is a member of the Cap‘n’Collar/basic-leucine zipper family of transcription factors [1]. Nrf2 governs the expression of genes containing a cis-acting regulatory element termed the antioxidant response element (ARE). Many ARE-driven genes are antioxidant and phase II detoxifying enzymes, thus Nrf2 activation can prevent or reduce cellular damage associated with several types of injury in many different tissues and organs [2]–[5]. Genetic disruption of Nrf2 aggravates neuronal death in models of Huntington’s [6] and Parkinson’s diseases [7]–[9], while Nrf2/ARE activation specifically in astrocytes confers protection to neighboring neurons in culture and *in vivo* [8], [10]–[12].

Amyotrophic lateral sclerosis (ALS) is the most common adult-onset motor neuron disease, caused by the progressive degeneration of motor neurons in the spinal cord, brain stem, and motor cortex. Motor neuron death leads to muscle weakness and paralysis causing death in one to five years from the time of symptom onset [13]. Approximately 10%–20% of familial ALS cases are caused by a toxic gain-of-function induced by mutations of the Cu/Zn-superoxide dismutase (SOD1) [14]. Over 150 mutations in the *SOD1* gene have been identified in familial ALS [15]. Rodents overexpressing mutated forms of hSOD1 generally develop an ALS-like phenotype [16], [17]. Several hypotheses, including oxidative stress, glutamate excitotoxicity, formation of high molecular weight aggregates, and mitochondrial dysfunction have been proposed to explain the toxic effect of mutant SOD1 [18]–[21]. The study of fALS-linked mutant hSOD1 animal models led to the development of the concept that the toxicity of mutant hSOD1 is not restricted to motor neurons but involves other cell types and thus, motor neuron fate depends on the interplay between different cell types [22].

Both Nrf2 and ARE-driven genes are upregulated and co-localize with reactive astrocytes in the spinal cord of symptomatic hSOD1^G93A^ animals [23], [24]. However, a reduction in Nrf2 expression has been reported in neurons from primary motor cortex and spinal cord from ALS postmortem tissue samples [25], and a similar decrease in the mRNA encoding Nrf2 was observed in embryonic motor neurons isolated from hSOD1^G93A^ rats [26]. In the ALS-afflicted muscle there is a gradual and selective loss of a subgroup of fast-type (type II) neuromuscular synapses while slow-type (type I) synapses resisted up to the terminal phase of the disease [27], [28]. Interestingly, we have shown that type I fibers display an increase in Nrf2 dependent transcription before symptoms onset in hSOD1^G93A^ mice, suggesting that this increase may be linked to their resistance to denervation [29].

We have previously shown that ALS mice with specific Nrf2 overexpression in astrocytes developed the disease later, survived longer, and had lower glial activation [12]. Here we sought to determine the effects of the absence of Nrf2 and its selective overexpression in neurons or type II skeletal muscle fibers on the course of the disease in ALS animal models.

## Materials and Methods

### Animals

B6.Cg-Tg(SOD1*G93A)1Gur/J [16], B6.Cg-Tg(SOD1*G85R)148Dwc/J [30] and FVB/N-Tg(Thy1-cre)1Vln/J [31] were obtained form The Jackson Laboratory (Bar Harbor, ME). Nrf2 knockout [32], ARE-hPAP mice [33] and caNrf2 transgenic [34] mice were previously described. To generate the MLC-Nrf2 transgenic mice, the mouse *Nrf2* gene was placed under the control of the type II muscle fiber-specific promoter for the myosin light chain 1 (MLC1). A SalI-EcoRI (1.8kb) SV40 promoter and enhancer fragment was inserted into a plasmid containing an MLC1 promoter [35] followed by the insertion of a NotI-NotI (2.4kb) fragment from mouse Nrf2. The fragment for microinjection was excised with Sal I, isolated by agarose gel electrophoresis and purified using GeneClean Turbo Kit (MP Biomedicals, Solon, OH). Transgenic mice were generated by pronuclear microinjection using fertilized eggs of the FVB/N strain. Five founder mice were identified by PCR. All experiments reported here utilized mice from the highest expressing line denominated #888. All the transgenic lines employed were on a C57BL/6J background, except for the Thy1-Cre and the MLC-Nrf2, which were on FVB. For the lifespan studies in MLC-Nrf2(+)/hSOD1^G93A^ mice, B6.Cg-Tg(SOD1*G93A)1Gur/J males were mated with FVB/N.MLC-Nrf2 females and onset and survival was determined in F1 animals. In this way, although in a mixed background, the contribution of each background was maintained constant throughout the study. Following genotyping animals were tagged and randomly caged, subsequent analyses were performed by an observer blinded to the genotype. End-stage was determined by the inability of the animal to right itself within 20 seconds when placed on its side. This is a widely accepted and published endpoint for life span studies in ALS-mice and guarantees that euthanasia occurs prior to the mice being unable to forage for food or water. Mice that were unable to right themselves within 20 seconds were euthanized immediately and recorded as dead for the purpose of life span studies. Animals were euthanized by CO_2_ asphyxiation and death was confirmed by verifying respiratory arrest. Mice were weighed two times per week and disease onset was retrospectively determined as the time when mice reached peak body weight. All animal procedures complied with the Animal Care and Use Committee requirements of the University of Wisconsin-Madison (Laboratory Animal Welfare Public Health Service Assurance Number: A3368-01). The UW-Madison IACUC approved all the procedures in this study.

### Cell cultures and treatment

Primary neuronal cultures were prepared from E15 cortices as previously described [33] with minor modifications. Cells were plated at a density of 1.5×10^5^ cells/cm^2^ and maintained in Neurobasal medium supplemented with B27 and 0.5 mM Glutamine (Invitrogen Carlsbad, CA). Cells were harvested or treated on the fourth day after plating. Cultures were >98% pure as judge by βIII-tubulin and GFAP staining. Treatments were performed in Neurobasal medium supplemented with B27 Minus AO (Invitrogen). Hydrogen peroxide (H_2_O_2_) was diluted in Dulbecco’s phosphate-buffered saline and applied to the cultures at the indicated final concentrations. Survival was assayed 24hs later by determining the release of lactate dehydrogenase (LDH) with the CytoTox Non-Radioactive Cytotoxicity Assay kit (Promega, Madison, WI).

### Real-time PCR

Total RNA was isolated using TRIZol reagent (Invitrogen). RNA quality was assessed with the 2100 Bioanalyzer (Agilent Technologies, CA, USA) and 2 μg were randomly reverse transcribed using SuperScript II reverse transcriptase (Invitrogen) according to the manufacturer’s protocol. PCRs were carried out in a 20 μl reaction with 1X LightCycler480 SYBR Green I Master (Roche, Indianapolis, IN) containing 1 μl of cDNA and 20 pmoles of each specific primer in a LightCycler480 Real-time PCR System (Roche). The cycling parameters were as follows: 95 °C, 10 s; 55 °C, 10 s; 72 °C, 15 s. Minus reverse transcriptase controls were included in each assay. Primers for *Nrf2*, *Nqo1*, *Ho-1*, *Gclm*, *Gclc* and *β-actin* were previously described [12]. Specific primers for *Keap1* were as follow: 5′-TTAAAGCCATGTTCACCAACGGGC-3′ and 5′-AGGCCGTGTAGGCGAACTCAATAA-3′.

### Glutathione measurement

Total glutathione levels (GSH and GSSG) were determined using the Tietze method as previously described [36]. Cells were lysed with ice-cold 3% perchloric acid while tissues were lysed in 5 volumes of 5% sulfosalicylic acid. Glutathione content was corrected by protein concentration determined by BCA protein assay (Thermo Scientific, Rockford, IL).

### Placental alkaline phosphatase histochemistry

Mice were transcardially perfused with 0.1 M PBS, followed by 4% paraformaldehyde in PBS (pH7.4). Spinal cord and muscles were dissected and cryoprotected in 30% and 20% sucrose-PBS respectively. Muscles were flash-frozen in isopentane. hPAP histochemistry was performed in 20μm cryostat sections as described [33].

### Statistical analysis

Each experiment was performed in duplicate and repeated at least three times. Groups of at least three animals were used for biochemical analysis and all data are reported as mean ± SD. Survival and onset data was analyzed with Kaplan-Meier curves and log rank test. Multiple group comparison was performed by one-way ANOVA with Bonferroni’s post-test, when comparing the effect of genotype and treatments two-way ANOVA was used followed by Bonferroni’s post-test and differences were declared statistically significant if *p* < 0.05. All statistical computations were performed using GraphPad Prism 4.0 (GraphPad Software, San Diego, CA).

## Results

### Nrf2 knockout

**Figure 1:**
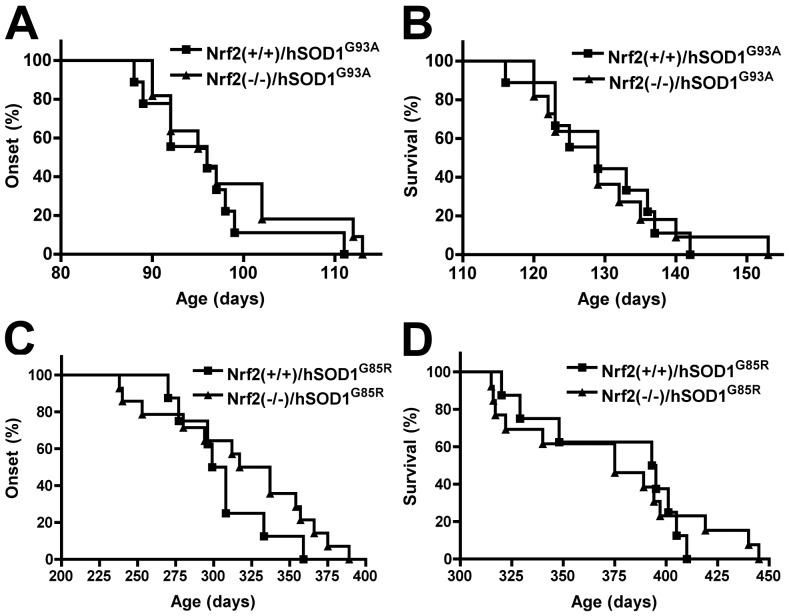
Absence of Nrf2 does not exacerbate neurodegeneration in mutant hSOD1 mice. A) Median onset in Nrf2(+/+)/hSOD1^G93A^ (96 days, n = 9) and Nrf2(-/-)/hSOD1^G93A^ mice (96 days, n = 11). B) Median survival in Nrf2(+/+)/hSOD1^G93A^ (129 days, n = 9) and Nrf2(-/-)/hSOD1^G93A^ mice (129 days, n = 11). Onset and survival curves are not significantly different. C) Median onset in Nrf2(+/+)/hSOD1^G85R^ (303.5 days, n = 8) and Nrf2(-/-)/hSOD1^G85R^ mice (327 days, n = 14). B) Median survival in Nrf2(+/+)/hSOD1^G85R^ (394 days, n = 8) and Nrf2(-/-)/hSOD1^G85R^ (375 days, n = 13). Onset and survival curves are not significantly different.

To determine the relevance of the reduced Nrf2 expression observed in motor neurons from ALS patients and animal models [25],[26] we analyzed whether the lack of Nrf2 activity would aggravate motor neuron degeneration in ALS mice. We employed Nrf2 knockout mice generated by the disruption of the DNA-binding domain of Nrf2 [32]. Since different hSOD1 mutants differ in important biochemical properties, including the capacity to dismutate superoxide, we investigated the effect of the lack of Nrf2 activity in two animal models of ALS-linked mutant hSOD1 overexpression. The widely used hSOD1^G93A^ model retains dismutase activity while the hSOD1^G85R^ has minimal or no dismutase activity. Disease onset and median survival was not significantly different when comparing Nrf2(+/+)/hSOD1^G93A^ with Nrf2(-/-)/hSOD1^G93A^ animals (Figure 1A,B). The lack of effect of Nrf2 deficiency was also observed in hSOD1^G85R^ mice in a Nrf2(-/-) background (Figure 1C,D).

### Neuron-restricted Nrf2 overexpression

Albeit the lack of Nrf2 activity did not aggravate the phenotype of hSOD1 mutant mice, we decided to test whether increasing Nrf2 activity selectively in neurons could have a protective effect. Under basal conditions, Nrf2 is constantly ubiquitinated by the Cul3–Keap1 ubiquitin E3 ligase complex and rapidly degraded by the proteasome [4]. Deletion of the Neh2 domain in Nrf2 eliminates the Keap1 binding site and generates a constitutively active transcription factor (caNrf2). We used a previously described transgenic mouse model in which the cDNA for caNrf2 was cloned into an expression cassette with a β-actin promoter and behind a transcription/translation STOP cassette flanked by *loxP* sites [34]. To induce Nrf2 expression selectively in neuronal cells, caNrf2 mice were mated with transgenic mice expressing Cre recombinase under control of the mouse Thy1 gene promoter (Thy1.2 sequence) [31]. Therefore, the caNrf2 overexpressed in neurons escapes the negative control of endogenous Keap1.

**Figure 2:**
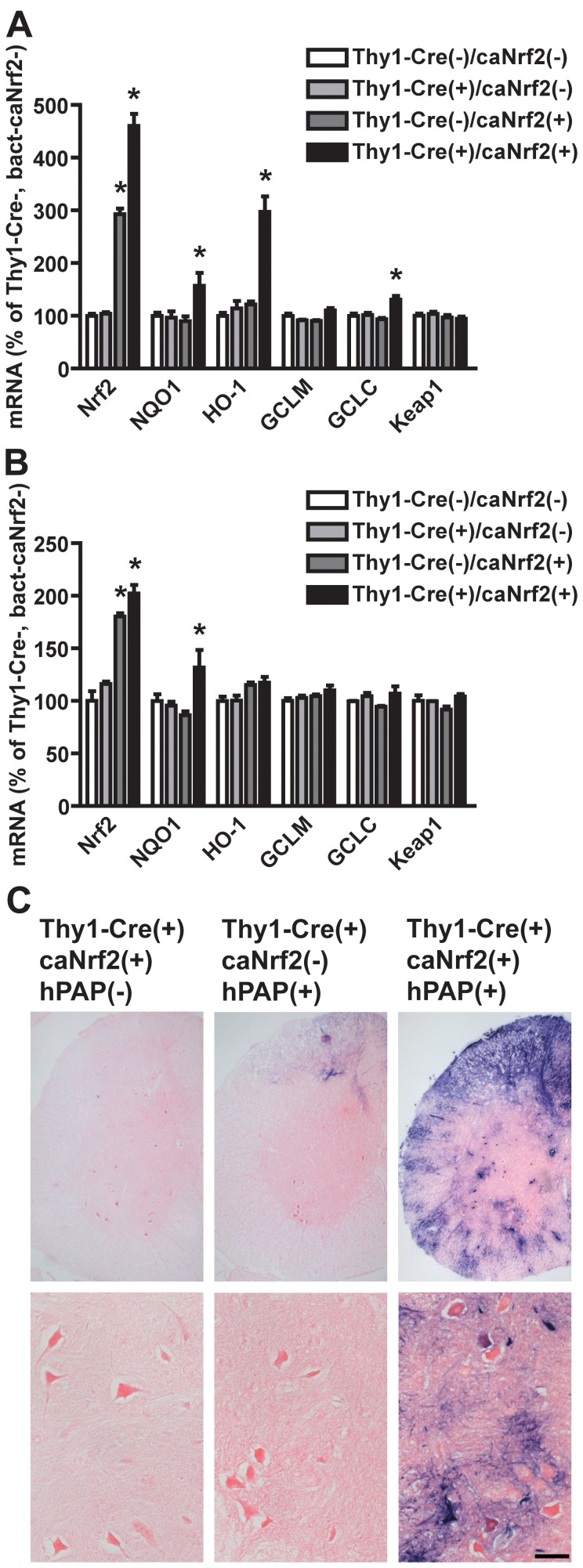
Overexpression of ARE-driven genes in Thy1-Cre(+)/caNrf2(+) mice. Increased mRNA expression levels of Nrf2 and ARE-driven genes in the cortex (A) and spinal cord (B) from 30 days old animals of the indicated genotypes. *Nrf2*, *Nqo1*, *Ho-1*, *Gclm*, *Gclc* and *Keap1* mRNA levels were determined by real-time PCR and corrected by *β-actin* mRNA levels. No difference in *Keap1* mRNA levels was observed. Data are expressed as mean±SD (n≥3). *Significantly different from Th1-Cre(-)/caNrf2(-) (p≤0.05). C) hPAP histochemistry in lumbar spinal cord sections from 30 days old mice of the indicate genotypes. Scale bar: 50 µM.

Nrf2 overexpression in Thy1-positive cells increased the transcription of prototypical ARE-driven genes, including NADPH:quinone oxidoreductase 1 (*Nqo1*), heme oxygenase 1 (*Ho-1*) and the catalytic subunit of the glutamate-cysteine ligase (*Gclc*), in the cortex of Thy1-Cre(+)/caNrf2(+) mice (Figure 2A). The increase in *Nrf2* mRNA observed in Thy1-Cre(-)/caNrf2(+) animals does not produce an increase in Nrf2 protein or ARE-driven genes expression (Figure 2A). This increase in *Nrf2* mRNA is due to the presence of the *caNrf2* transgene, but the messenger is not translated, since the STOP cassette primarily inhibits translation. At the spinal cord level we only detected an increase in *Nrf2* and *Nqo1* mRNA levels (Figure 2B). This is probably due to a smaller ratio of neurons to glial cells in the spinal cord compared to cortex, and its dilution effect on total RNA. The upregulation of Nrf2 activity in the spinal cord was confirmed by breeding the Thy1-Cre(+)/caNrf2(+) animals with mice expressing the human placental alkaline phosphatase (hPAP) driven by a promoter containing a consensus ARE sequence [ARE-hPAP, [33]]. A significant increase in hPAP histochemical staining in spinal cord motor neurons was observed in Thy1-Cre(+)/caNrf2(+) mice (Figure 2C).

**Figure 3:**
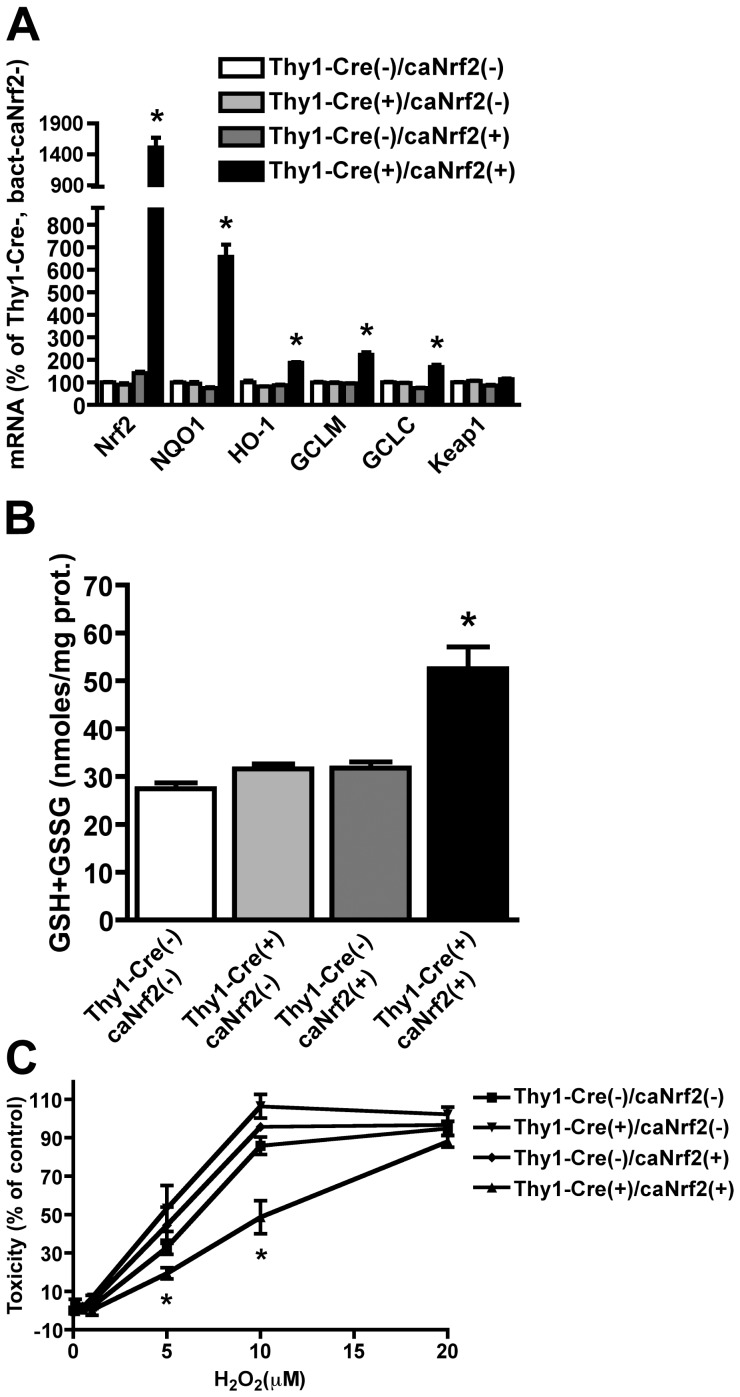
Increased mRNA expression of *Nrf2* and ARE-driven genes in cortical neurons increases glutathione content and resistance to oxidative stress. A) Total RNA was extracted from pure neuronal cultures and *Nrf2*, *Nqo1*, *Ho-1*, *Gclm*, *Gclc* and *Keap1* mRNA levels were determined by real-time PCR and corrected by *β-actin* mRNA levels. mRNA levels are expressed as percentage of Thy1-Cre(-)/caNrf2(-) neurons. B) Increased total glutathione (GSH+GSSG) content in Thy1-Cre(+)/ca-Nrf2(+) cortical neurons. C) Neuronal cultures were treated with the indicated concentrations of H_2_O_2_ and toxicity was determined 24hs later by LDH release assay. Data are expressed as percentage of their respective control. A, B and C, Data are expressed as mean±SD (n≥3). *Significantly different from Thy1-Cre(-)/caNrf2(-) (p≤0.05).

Primary neuronal cultures obtained from Thy1-Cre(+)/caNrf2(+) mice display approximately a 14-fold increase in Nrf2 mRNA that correlates with a significant increase in *Nqo1*, *Ho-1*, *Gclm* and *Gclc* mRNA (Figure 3A). GCLM and GCLC are the rate-limiting enzymes in glutathione synthesis and the increase in *Gclm* and *Gclc* expression correlated with almost a 2-fold increase in total glutathione levels in Thy1-Cre(+)/caNrf2(+) cultures when compared to controls (Figure 3B). Accordingly, Thy1-Cre(+)/caNrf2(+) neurons displayed increased resistance to oxidative stress as reflected by reduced vulnerability to hydrogen peroxide toxicity (Figure 3C).

**Figure 4:**
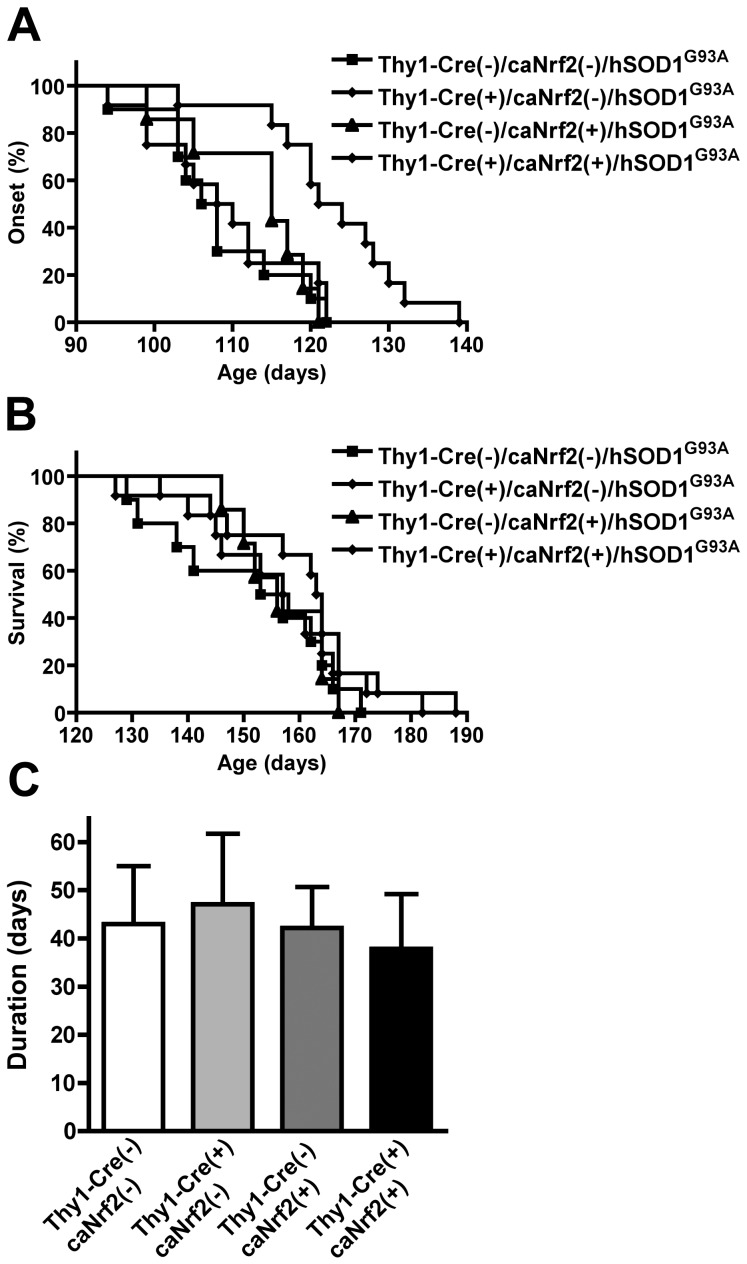
Selective overexpression of Nrf2 in neurons delays onset but does not extend survival in hSOD1^G93A^ mice. A) Median onset in Thy1-Cre(-)/caNrf2(-)/hSOD1^G93A^ (107 days, n = 10), Thy-1Cre(+)/caNrf2(-)/hSOD1^G93A^ (109 days, n = 12), Thy1-Cre(-)/caNrf2(+)/hSOD1^G93A^ (115 days, n = 7) and Thy1-Cre(+)/caNrf2(+)/hSOD1^G93A^ mice (122.5 days, n = 12). Onset curves are significantly different (p≤0.05, χ^2^ = 10.6). B) Median survival in Thy1-Cre(-)/caNrf2(-)/hSOD1^G93A^ (155 days, n = 10), Thy-1Cre(+)/caNrf2(-)/hSOD1^G93A^ (157.5 days, n = 12), Thy1-Cre(-)/caNrf2(+)/hSOD1^G93A^ (156 days, n = 7) and Thy1-Cre(+)/caNrf2(+)/hSOD1^G93A^ mice (163.5 days, n = 12). Survival curves are not significantly different. C) Disease duration (time from onset to death). All animals are hSOD1^G93A^ positive. Data are expressed as mean±SD (n as for A and B).

To investigate the effect of selective neuronal Nrf2 overexpression in the survival of hSOD1^G93A^ mice, we generated Thy1-Cre(+)/caNrf2(+)/hSOD1^G93A^ triple-transgenic mice. The median disease onset as reflected by body weight loss was significantly delayed by approximately 7 days when Thy1-Cre(-)/caNrf2(+)/hSOD1^G93A^ animals were compared to Thy1-Cre(+)/caNrf2(+)/hSOD1^G93A^ mice (Figure 4A). Although not statistically significant, survival was extended also by 7 days in the triple-transgenic mice [Thy1-Cre(-)/caNrf2(+)/hSOD1^G93A^ (156 days, n = 7) and Thy1-Cre(+)/caNrf2(+)/hSOD1^G93A^ mice (163.5 days, n = 12), Figure 4B] and disease duration was not significantly modified (Figure 4C).

### Skeletal muscle-restricted Nrf2 overexpression

In addition to motor neurons, the skeletal muscle might also be a target of mutant SOD1-mediated toxicity [37]. In ALS-afflicted muscles, type I fibers appear to maintain synaptic connections with motor neurons far after type II fibers have been denervated [27], [28]. We have previously shown that during the course of the pathology in hSOD1^G93A^ mice, the fibers that display the earliest and strongest Nrf2 activation (ARE-hPAP enzymatic activity) are type I fibers rather than type II fibers [29], suggesting that this increase may be linked to their resistance to denervation. To test whether an increase in Nrf2 activity in type II fibers could confer protection, we developed transgenic mice that overexpress Nrf2 under the control of the myosin light chain 1 (MLC1) promoter (MLC-Nrf2), a protein selectively expressed in type II fibers [35].

**Figure 5:**
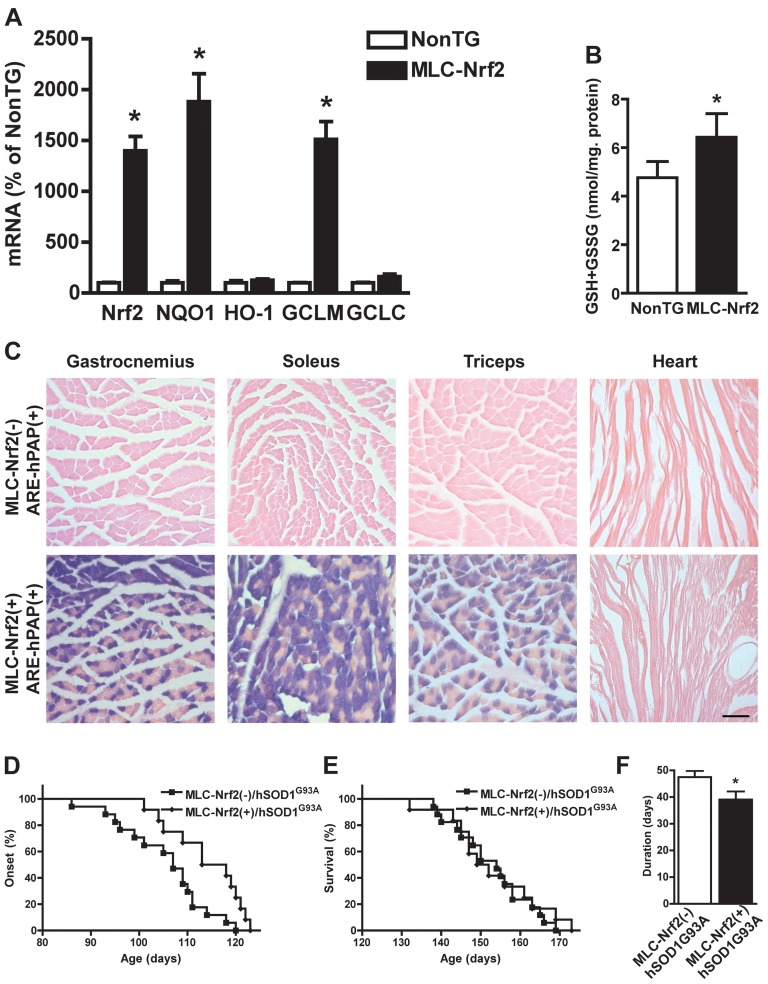
Selective overexpression of Nrf2 in type II muscle fibers delays onset but does not extend survival in hSOD1^G93A^ mice. A) Increased mRNA expression of Nrf2 and ARE-driven genes in the gastrocnemius muscle from 30 days old non-transgenic (NonTG) and MLC-Nrf2 mice. *Nrf2*, *Nqo1*, *Ho-1*, *Gclm* and *Gclc* mRNA levels were determined by real-time PCR and corrected by *β-actin* mRNA levels. Data are expressed as mean±SD (n≥3). *Significantly different from NonTG (p≤0.05). B) Increased total glutathione (GSH+GSSG) content in the gastrocnemius muscle from 30 days old NonTG and MLC-Nrf2 mice. Data are expressed as mean±SD (n≥3). *Significantly different from NonTG (p≤0.05). C) hPAP histochemistry in muscle sections from 30 days old mice of the indicate genotypes. Scale bar: 50 µM. D) Median onset in MLC-Nrf2(-)/hSOD1^G93A^ (107 days, n = 17) and MLC-Nrf2(+)/hSOD1^G93A^ (115.5 days, n = 12) mice. Onset curves are significantly different (p≤0.01, χ^2^ = 6.7). E) Median survival in MLC-Nrf2(-)/hSOD1^G93A^ (154 days, n = 17) and MLC-Nrf2(+)/hSOD1^G93A^ (150.5 days, n = 12) mice. Survival curves are not significantly different. F) Disease duration (time from onset to death). All animals are hSOD1^G93A^ positive. Data are expressed as mean±SD (n as for D and E).

In the gastrocnemius muscle from MLC-Nrf2 mice, Nrf2 overexpression induced a significant increase in the transcription of prototypical ARE-driven genes, like *Nqo1* and *Gclm*, as well as an approximately 35% increase in total glutathione content (Figure 5A, B). As reflected by hPAP histochemistry in MLC-Nrf2(+)/ARE-hPAP(+) mice, Nrf2 mediated transcriptional activation was significantly increased in several skeletal muscles that display mixed fiber type content, but not in cardiac muscle (Figure 5C). Overexpression of Nrf2 in type II fibers delayed the median onset of the disease by 8.5 days in MLC-Nrf2(+)/hSOD1^G93A^ mice when compared to controls (Figure 5D). However, the median survival was not significantly extended in MLC-Nrf2(+)/hSOD1^G93A^ mice (Figure 5E). The disease duration was significantly reduced from 47.5±2.3 days in MLC-Nrf2(-)/hSOD1^G93A^ mice to 39.1±3.0 in MLC-Nrf2(+)/hSOD1^G93A^ animals (p<0.05; Figure 5F).

## Discussion

Despite the dramatic effect that the knockout of Nrf2 has in many acute models of neuronal damage [6]–[9], we did not observe any detrimental effect associated with the absence of Nrf2 activity in two animal models of ALS. In addition, selective Nrf2 overexpression in neurons or type II skeletal muscle fibers delayed disease onset, but failed to extend the survival of hSOD1^G93A^ mice.

The lack of effect of the Nrf2 knockout in the context of chronic neurodegeneration may be explained in part by intrinsic characteristics of Nrf2 regulated transcription. While the induction of ARE-driven genes under stress conditions depends almost exclusively on *de novo* gene transcription, their constitutive expression may not critically depend on Nrf2 activity [3]. Such is the case of the enzymes related to glutathione synthesis: *Gclc* and *Gclm* [38]. In agreement, we found that the amount of total glutathione in the cortex, spinal cord and gastrocnemius muscle does not differ between Nrf2(+/+) and Nrf2 (-/-) mice (data not shown). As has been shown in many different models, increased levels of glutathione seems to be a major component of the protection conferred by Nrf2 activation [2]. Therefore, in response to an acute insult the absence of Nrf2 may be critical, but in the context of a chronic pathology cells may have redundant systems to compensate for the lack of Nrf2 activity, thus explaining the lack of effect in these ALS mouse models.

While in the central nervous system ARE-driven genes are preferentially activated in astrocytes [10], [11], our results of Nrf2 overexpression in Thy1-positive cells show that neurons indeed have a reserve capacity to up-regulate the transcription of ARE-driven genes and directly increase glutathione synthesis. *In vitro*, this increase in antioxidant defenses was able to confer protection against a toxic oxidative insult and adds to the current paradigm of Nrf2 as a potential therapeutic target. However, in hSOD1^G93A^ mice, neuronal overexpression of Nrf2 was only able to marginally delay onset. This may suggest that, in the context of the ongoing neurodegenerative process, neurons are not able to maintain a sustained Nrf2 activation, or that the protective response might temporally stall neuronal degeneration in early stages of the disease. However, this effect is eventually overwhelmed by the pathogenic mechanisms. Also, motor neuron degeneration in ALS depends at least in part on the damage or dysfunction of non-neuronal cells [22], and the selective increase of Nrf2 activity in neurons might not be effective against the toxic properties of ALS glial cells.

Although, the role of mutant hSOD1 toxicity in muscle remains controversial [37], [39], therapies targeting muscle are attractive due to easier accessibility compared to the nervous system. Type II muscle fiber-restricted Nrf2 overexpression effectively increased ARE-driven genes and total glutathione content in muscle cells and delays symptoms onset in hSOD1^G93A^ animals. However, no significant extension in survival was observed since the disease duration was significantly shortened. This suggests that an increase in antioxidant defenses in the skeletal muscle could contribute to delay muscle wasting by preserving a functional neuromuscular junction, even after thinning of the nerve terminals (around p30-p40) and sprouting (p60) is typically observed in the hSOD1^G93A^ mouse model [27], [28]. Since, muscle Nrf2 overexpression does not centrally affect the pathological process, once the complete denervation occurs no other beneficial effect of Nrf2 overexpression is observed and the MLC-Nrf2(+)/hSOD1^G93A^ mice display the same median survival than the controls.

According to the current notion of non-cell autonomous toxicity in ALS [40], [41], a modification that directly affects motor neurons will delay the onset, while modifications of the glial compartment will predict a shift in progression. In both transgenic approaches Nrf2 overexpression delayed onset without affecting the overall survival. This may reflect the fact that Nrf2 overexpression in neurons and muscle cells cannot delay glial activation and thus account for the lack of effect in progression and overall survival.

While we showed here that selective Nrf2 overexpression in neurons or type II muscle fibers only delay onset, we have previously shown that ALS-mice with specific astrocytic Nrf2 overexpression developed the disease later and survived longer [12]. These results suggest the fundamental role of astrocytes in determining neuronal antioxidant defenses and highlight the concept that not only the pharmacological target but also the cell type targeted may be relevant when considering an Nrf2-dependent therapeutic approach for ALS.
