# Correction: Absence of Nrf2 or Its Selective Overexpression in Neurons and Muscle Does Not Affect Survival in ALS-Linked Mutant hSOD1 Mouse Models

**DOI:** 10.1371/annotation/28f68b10-e23d-4519-8d52-8cc94fe372b1

**Published:** 2013-04-16

**Authors:** Marcelo R. Vargas, Neal C. Burton, Li Gan, Delinda A. Johnson, Matthias Schäfer, Sabine Werner, Jeffrey A. Johnson

Author Jennifer Kutzke should be included in the Author Byline, listed third, and her affiliation should be 1: Division of Pharmaceutical Sciences, University of Wisconsin, Madison, Wisconsin, United States of America

The contributions of this author are as follows: Performed the experiments, analyzed the data.

The correct citation is: Vargas MR, Burton NC, Kutzke J, Gan L, Johnson DA, et al. (2013) Absence of Nrf2 or Its Selective Overexpression in Neurons and Muscle Does Not Affect Survival in ALS-Linked Mutant hSOD1 Mouse Models. PLoS ONE 8(2): e56625. doi:10.1371/journal.pone.0056625 

